# Single-center experience in the treatment of extremely medial clavicle fractures with vertical fixation of double-plate: A retrospective study

**DOI:** 10.1097/MD.0000000000019605

**Published:** 2020-04-03

**Authors:** He Liu, Chuangang Peng, Ziyan Zhang, Baoming Yuan, Guangkai Ren, Junlong Yu, Dankai Wu

**Affiliations:** a Department of Orthopedics, The Second Hospital of Jilin University, Changchun; b Department of Orthopedics, Rushan People's Hospital, Weihai, China.

**Keywords:** double-plate, extremely medial clavicle fracture, vertical fixation

## Abstract

Patients suffering from extremely medial clavicle fractures combined with distinct displacement generally need surgical intervention. Double-plate fixation is a widely applied technique in the treatment of distal radius fracture, which has been reported to fix lateral clavicle fracture as well. This study reveals the effect of double-plate fixation as an innovative procedure in the treatment of extremely medial clavicle fractures for the first time.

Nine patients complaint of extremely medial clavicle fracture were enrolled in this research from May 2017 to March 2019. Patients were operated with an open reduction and internal fixation using the double-plate technique. Postoperative x-ray was taken regularly to observe the fracture healing at each visit, and the related complications were also recorded. The rating score systems of Constant Murley score of treated shoulder and contralateral shoulder, ROWE score as well as American Shoulder and Elbow Surgeons (ASES) were evaluated to comment on the postoperative shoulder joint function.

All patients achieved postoperative fracture healing with no complications. Only 1 patient complained of slight restriction, 2 patients complained of pain during overhead work, and another patient was found with plate breakage. Meanwhile, the Constant Murley scores of treated and contralateral shoulder were 94.1 and 98.5 points, respectively, indicating the similar shoulder function. Furthermore, the ROWE and ASES scores of the involved shoulder were 96.7 and 96.3 points at average, respectively.

It is the first time to introduce the surgical technique of vertical double-plate fixation implied in stable fixation of extremely medial clavicle fractures, which could provide the surgeons with an alternative method for this type of fracture.

## 1 Introduction

The extremely medial clavicle fracture refers to the fracture locating at the 1/5 position of the medial clavicle, with or without sternoclavicular joint dislocation, accounting for 3% to 6% of the clavicular fracture.^[1]^ General conservative treatment can achieve considerable curative effect, which is commonly feasible for type I A fracture in the Edinburgh classification. While patients accompanied by obvious displacement, sternoclavicular joint dislocation, or vascular and nerve injury, the surgical treatment is generally demanded.^[2]^

Kirschner-wire and tension band are conducive to fracture healing and reliable fixation, which can facilitate the early functional exercise of the shoulder and the recovery of joint function. However, the tension strength of the Kirschner-wire and tension band is instable, thus it is prone to cause re-fracture, dislocation, loosening, withdrawal, and migration as well as vascular injury and nerve compression.^[3,4]^ T shaped locking plate fixation is recognized to be used for the treatment of clavicle fractures, which is fixed by a single cortex to avoid damage to adjacent tissues. While the fixation is poor, early postoperative functional exercise may result in stress concentration causing the plate loosening or re-fracture.^[5]^ The clavicular hook plate is another routine procedure, but characterized with poor resistance to twisting and rotation, which is easy to break through the sternum. It is difficult to remove the hook plate, and then it may damage the sternoclavicular joint.^[6]^ Additionally, reconstruction plate is an alternative implant, but it should be careful to avoid damage to the subclavian arteries, veins and brachial plexus, penetrating the mediastinum and pleura, to prevent from serious complications. However, because this kind of plate is thin and the clavicle is subjected to large shear force and stress shielding, appropriate external fixation may be needed after operation to avoid fixation failure and affect fracture healing.^[7]^

Double-plate fixation is a common technique in the treatment of complex fractures of the distal radius or ulna.^[8,9]^ Successful treatment of clavicle distal Neer II fractures has been confirmed with double fracture-fixation techniques, which can effectively improve the fixation stability.^[10]^ Furthermore, the biomechanical testing demonstrated that double plate technique was mechanically superior to the single plate technique in lateral clavicle fractures fixation.^[11]^ In this research, it is the first time to investigate the feasibility for the treatment of medial clavicle fractures using the double-plate fixation technique.

## 2 Ethical approval

The study was approved by the ethics committee of the Second Hospital of Jilin University. The enrolled patients were given written informed consent for this report, and their anonymity were kept confidential. (2019) Research and Inspection No. (060).

## 3 Patients and methods

### 3.1 Patients characteristics

**Figure 1:**
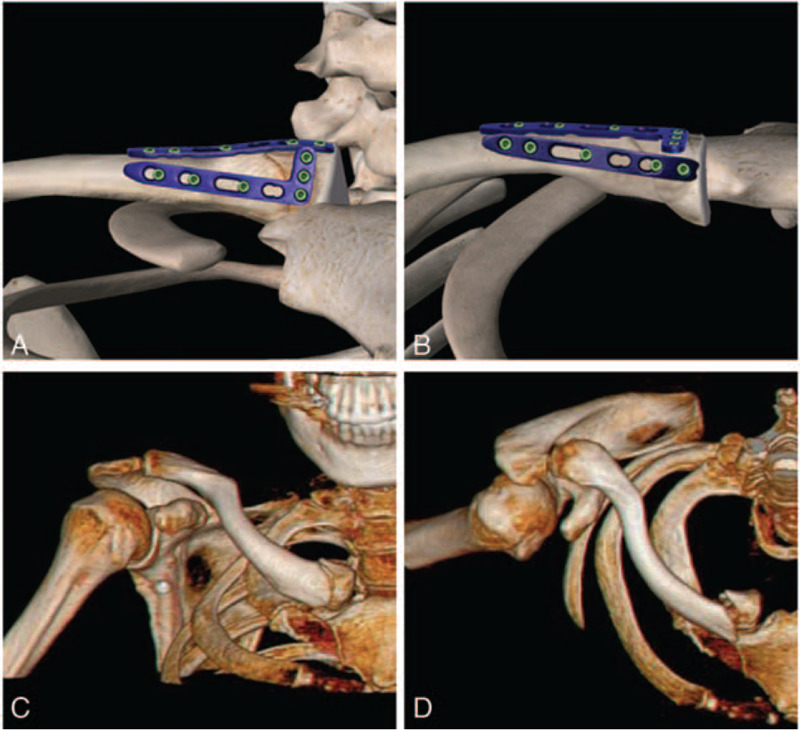
Front view (A, C) and top view (B, D) of the plate placement and the 3D reconstruction of a medial clavicle fracture in a female patient.

Extremely medial clavicle fracture was defined as clavicle fracture located at 2 cm within medial side (1/5 position of the medial clavicle). Nine patients with extremely medial clavicle fractures were treated with open reduction and internal fixation using the double-plate technique (Fig. 1A and B) from May 2017 to March 2019. X-ray examination and three dimensional (3D) reconstruction (Fig. 1C and D) were conducted in each patient. According to the Edinburgh Classification, among the 9 patients, 4 types of fracture were included: type A I (2 cases), type A II (2 cases), type B I (4 cases), and type B II (1 case). The general information of involved patients, such as sex, age, and fracture classification, is listed in Table 1.

**Table 1:**
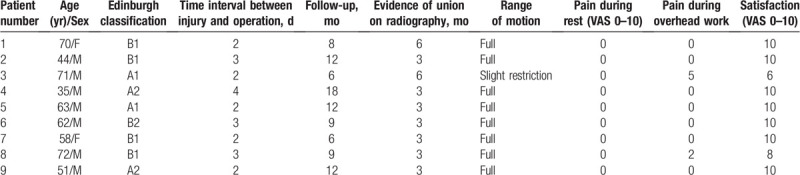
Basic information and follow-up observations of 9 patients treated with double-plate fixation for medial clavicle fractures.

### 3.2 Surgical technique

**Figure 2:**
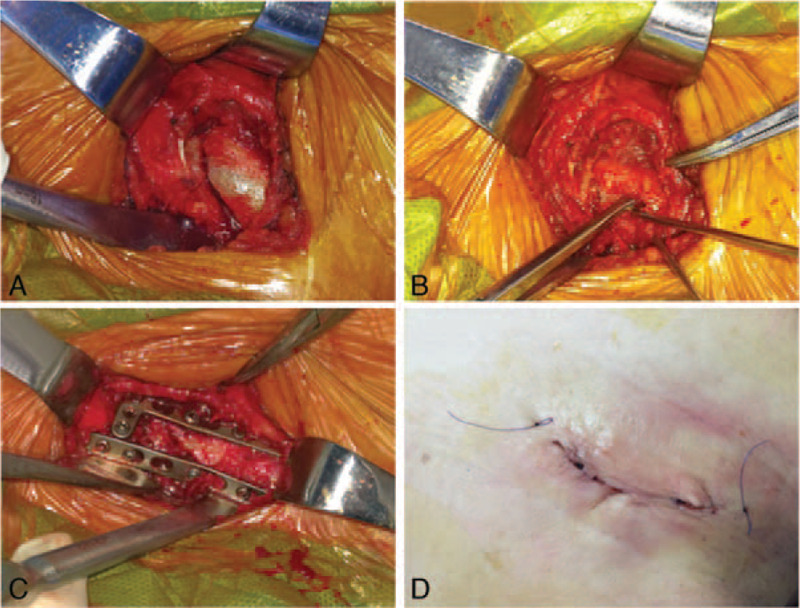
Operative views during double-plate fixation using 2 2.4/2.7 mm locking compression plates: surrounding soft tissue and incarcerated periosteum in the fracture gap were removed (A); Kirschner-wire was used for temporary fixation (B); two plates were placed vertically (C); image of the wound (D).

**Figure 3:**
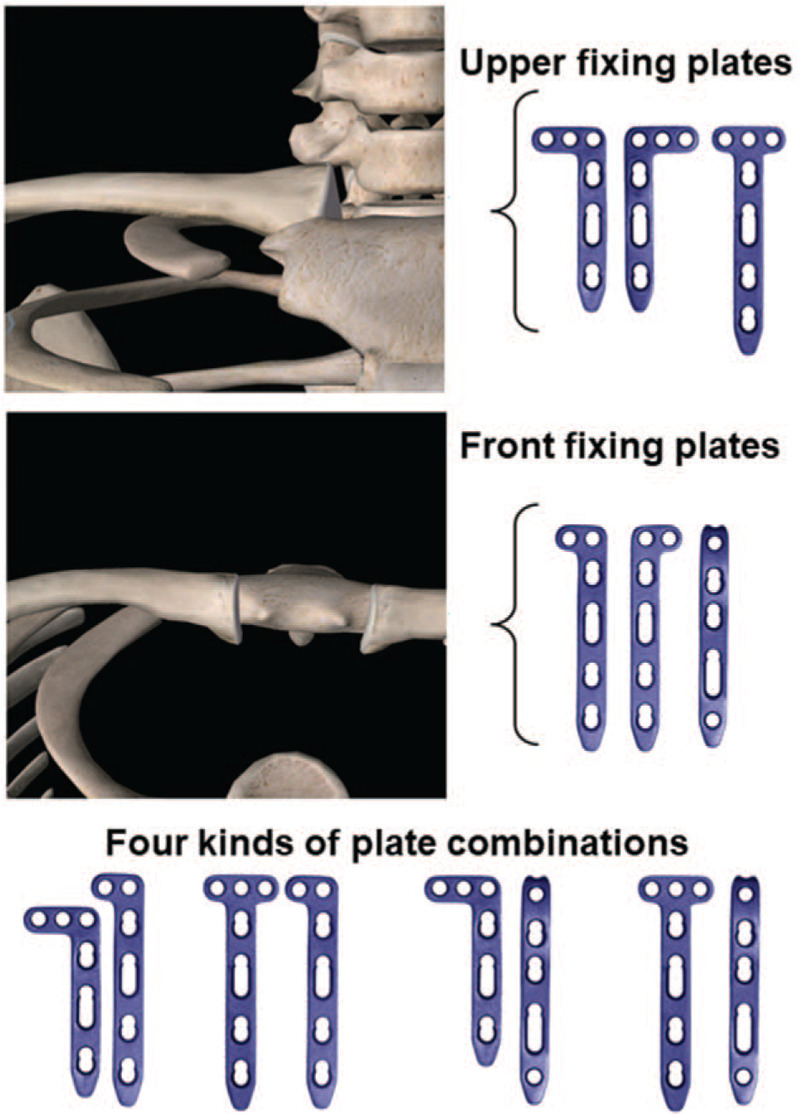
Four kinds of plate combination for medial clavicle fracture fixation using double-plate system.

After brachial plexus anesthesia, the patient was positioned in a beach chair position, and the injured shoulder was padded with clavicle fracture centered. The transverse incision above the clavicle was applied to expose the sternoclavicular joint. We removed the surrounding soft tissue and released the incarcerated periosteum from the fracture gap (Fig. 2A). Then, absorbable non-invasive suture for bundling or Kirschner-wire for temporary fixation was used to make sure the clavicular reduction and avoid shortening deformity, and the operation was also checked by x-ray examination (Fig. 2B). Subsequently, a 2.4/2.7 mm locking compression plate (BD-SIT, Beijing, China) was bent according to the anatomic position of the clavicle, and positioned on the cranial surface of the clavicle. The plate was fixed with 3 cortical screws inserting into the distal clavicle fragment. The second 2.4/2.7 mm locking compression plate was placed vertically to the first plate on the anterior surface of the clavicle (Fig. 2C). After the final x-ray examination, the incision was sutured layer by layer (Fig. 2D), and finally pressure bandages were twined around the wound. Additionally, the plate for medial clavicle fracture fixation was selected using double-plate system, and 4 kinds of plate combinations are listed in Fig. 3.

### 3.3 Postoperative treatment

Twenty four hours after operation, functional activities such as forearm rotation, flexion, and extension of the elbow joint were performed. One week after the operation, the upper limbs were able to perform a small range of flexion, extension, and shrug. Functional exercise could start at 2 weeks postoperatively and no weight-bearing activity was recommended at 6 weeks postoperatively. We conducted regular radiographic follow-up at 3, 6, 9, and 12 months after surgery. Postoperative x-ray was taken regularly to observe the fracture healing at each visit, and the related complications were recorded. Meanwhile, the rating score systems of Constant Murley score,^[12]^ ROWE score as well as American Shoulder and Elbow Surgeons (ASES) were evaluated at the end of follow-up to comment on postoperative shoulder joint function.^[13,14]^

## 4 Results

**Figure 4:**
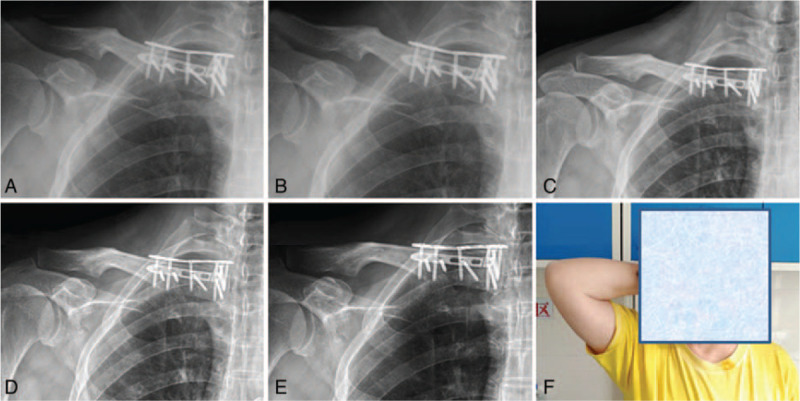
X-ray radiographs at 1 day (A), 3 months (B), 6 months (C), 9 months (D), and 12 months (E) of a female patient after double-plate fixation, as well as the shoulder joint extension (F) at 3 months postoperation.

All of the 9 patients were followed up for at least 6 months with an average of 10.2 months (from 6 to 18 months). X-ray images showed that all patients achieved postoperative fracture healing at an average of 3.6 months (from 3 to 6 months) (Fig. 4). After bone healing, no patient complained of pain during rest. There was mild pain in 2 patients’ affected side (22.2%) during overhead work (Visual Analogue Score 0–10, average score was 0.8). Overall, the mean satisfaction degree of all patients was 9.3 points. There was 1 patient (11.1%) reported that his shoulder internal rotation, abduction, and adduction were basically the same as the healthy side, and only the active external rotation was slightly restricted. Plate breakage was found in a patient, who did not adhere to the physician's advice and began heavy physical labor from 2 months after the operation. At the 3 months of follow-up, the plate was found with plate breakage, with no obvious uncomfortable feeling. At this time, x-ray examination showed that the fracture site was well healed. Plate removal was performed at 12 months after surgery. No other complications were founded, such as fractures around the plate, screw loosing, joint instability, and displacement. At the last follow-up, no complications, such as failure of internal fixation, nonunion, fractures around the plate, or traumatic arthritis, were recorded. (See Table 1)

Implants of 2 patients (22.2%) were removed at 12 and 18 months after surgery, respectively. All patients went back to work except for 3 patients who were already retired. Meanwhile, the Constant Murley scores of treated and contralateral shoulder were 94.1 and 98.5 points, respectively, indicating the similar shoulder function. Furthermore, the ROWE and ASES scores of the involved shoulder were 96.7 and 96.3 points at average, respectively. These rating score systems demonstrated good recovery of injured medial clavicle fractures collectively (Table 2).

**Table 2:**
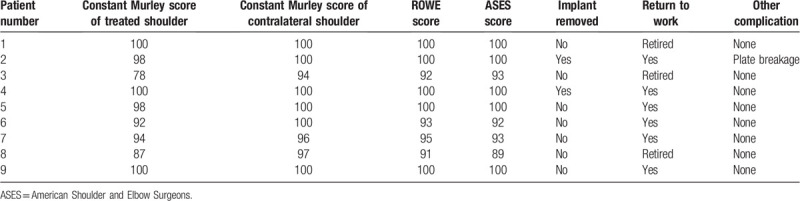
Functional assessments and clinical outcomes of 9 patients treated with double-plate fixation for medial clavicle fractures.

## 5 Discussion

Medial clavicle fractures often contain the rupture of sternoclavicular surface and/or ligament, mimicking an unstable fracture.^[15]^ As non-surgical treatment, it is difficult to obtain anatomical reduction. Nonoperative treatment may be complicated by delayed healing and high nonunion rate, although without anesthetic risk, osteomyelitis, vascular nerve injury, and other surgical complications.^[16]^ Therefore, surgical treatment should be performed as soon as possible if the general condition of the patient permits surgery. The purposes of the operation are to restrict the fracture, prevent shortening deformity, avoid malunion, and strengthen and fix the fracture firmly. The operation does not affect the activity of the sternoclavicular joint. As described previously, the traditional fixation methods have a high failure rate. The type of internal fixation for medial clavicle fixation is discussed controversially.

The advantages of double-plate fixation technique in the treatment of distal clavicle Neer II B fractures have been confirmed. Double-plate fixation technique can effectively improve the fixation effect.^[10,11]^ In this research, we chose 2 2.4/2.7 mm locking compression plates to vertically fix medial clavicle fracture with 4 to 6 locking screws. This double-plate fixation system possesses several advantages. Double-plate fixation could provide sufficient fixation, and fix even small fragments securely. This vertically arranged plate and screw construct could ensure the stable fixation of the medial clavicle end; double row locking screw fixation in different directions could promise more stable fixation. This theory has been verified by the biomechanical study that the opposing force of the screw in the front of the medial clavicle is the lateral shear force load, which could reduce the axial strength and stress shielding.^[17]^ Seven patients included were over 50 years old in this study, and no patients experienced nonunion and plate fixation failure, indicating a reliable fixation even for patients of older age. The sternoclavicular joint was not influenced in the screw fixation, and the activity of the sternoclavicular joint was not affected, so the shoulder joint function exercise could be started in the early postoperative period.

In addition to effectively reducing internal fixation failure, the double-plate technology could provide anatomical reduction and exhibited excellent bone healing with an average healing time for 3.6 months with minimal complications, such as plate breakage, slight restriction of motion range, and pain during overhead work. Stable fixation also provided favorable conditions for early functional exercise. The Constant Murley score of treated shoulder and contralateral shoulder was similar at the last follow-up. ROWE score and ASES score also indicated good recovery of shoulder function.

The strengths of using double-plate technique for the treatment of the medial clavicle fractures should be noted: avoid to cut and separate too much soft tissues in case of affecting the medial clavicle stability. For older patients, the operative action should be gentle and attention should be paid to avoid further crushing of the fracture. Meanwhile, early heavy physical labor should be strictly prohibited to avoid plate breakage. Anatomical reduction should be achieved, especially for the fracture in the sternoclavicular joint, to prevent the loss of clavicle length and the rotational deformity. The plate combinations should be selected according to the distance between the fracture and the sternoclavicular joint as well as the fracture type: a straight or 2-hole row plate could be used for upper fracture fixation, and a 3-hole L-shaped plate or a T-shaped plate could be used for front fracture fixation. The plates should be pre-shaped according to the shape of the clavicle to permit better attachment to the clavicle. At least 3 screws, usually 4 to 6 screws, should be used for fixing the medial clavicle fracture with rafting technique.

### 5.1 Study limitations

This is a relatively new technique with small number of cases involved. Meanwhile, there is the theoretical possibility of damaging the nerves and blood vessels behind the clavicle. Hence, our research needs to be confirmed by a larger randomized controlled study including preoperative assessment and conservatively treated control group.

## 6 Conclusion

Double-plate vertical fixation technique was investigated to evaluate the therapeutic effect on extremely medial clavicle fracture. The anatomical reduction could be achieved under direct vision with stable fixation, which avoided the interference with the sternoclavicular and acromioclavicular joint activity. The outcome of follow up showed good bone healing rate and shoulder joint functional recovery indicating satisfactory early curative effect.

## Acknowledgments

The authors would like to thank Prof. Jincheng Wang who provided encouragement to write this paper and gave valuable advice. Many thanks to reviewers for the constructive comments on earlier drafts.

## Author contributions

**Conceptualization:** He Liu, Chuangang Peng.

**Data curation:** Ziyan Zhang, Guangkai Ren, Junlong Yu.

**Writing – original draft:** He Liu, Baoming Yuan, Chuangang Peng.

**Writing – review & editing:** Dankai Wu.

He Liu orcid: 0000-0001-7178-0276.

## References

[1] Nowak J Mallmin H Larsson S The aetiology and epidemiology of clavicular fractures. A prospective study during a two-year period in Uppsala, Sweden. Injury 2000;31:353–8. 10775691 10.1016/s0020-1383(99)00312-5

[2] Kendal JK Thomas K Lo IKY Clinical outcomes and complications following surgical management of traumatic posterior sternoclavicular joint dislocations: a systematic review. JBJS Rev 2018;6:e2. 10.2106/JBJS.RVW.17.00157 30399119

[3] Reghine ÉL Cirino CCI Neto AA Clavicle Kirschnerwire migration into left lung: a case report. Am J Case Rep 2018;19:325–8. 29559613 10.12659/AJCR.908014 PMC5881454

[4] Lee YS Lau MJ Tseng YC Comparison of the efficacy of hook plate versus tension band wire in the treatment of unstable fractures of the distal clavicle. Int Orthop 2009;33:1401–5. 19050884 10.1007/s00264-008-0696-7 PMC2899132

[5] Kim KC Shin HD Cha SM Surgical treatment of displaced medial clavicle fractures using a small T-shaped plate and tension band sutures. Arch Orthop Trauma Surg 2011;131:1673–6. 21811810 10.1007/s00402-011-1367-5

[6] Gille J Schulz A Wallstabe S Hook plate for medial clavicle fracture. Indian J Orthop 2010;44:221–3. 20419013 10.4103/0019-5413.61768 PMC2856401

[7] Der Tavitian J Davison JN Dias JJ Clavicular fracture non-unionsurgical outcome and complications. Injury 2002;33:135–43. 11890915 10.1016/s0020-1383(01)00069-9

[8] Rikli DA Businger A Babst R Dorsal double-plate fixation of the distal radius. Eur J Trauma Emerg Surg 2007;33:99–109. 26815983 10.1007/s00068-007-9155-1

[9] Bessho Y Okazaki M Nakamura T Double plate fixation for correction of the malunited distal ulna fracture: a case report. Hand Surg 2011;16:335–7. 22072470 10.1142/S0218810411005643

[10] Kaipel M Majewski M Regazzoni P Double-plate fixation in lateral clavicle fractures-a new strategy. J Trauma 2010;69:896–900. 20093980 10.1097/TA.0b013e3181bedf28

[11] Suter C Majewski M Nowakowski AM Comparison of 2 plating techniques for lateral clavicle fractures using a new standardized biomechanical testing setup. J Appl Biomater Funct Mater 2018;16:107–12. 28885665 10.5301/jabfm.5000377

[12] Constant CR Murley AH A clinical method of functional assessment of the shoulder. Clin Orthop Relat Res 1987;214:160–4. 3791738

[13] Rowe CR Patel D Southmayd WW The bankart procedure: a long-term end-result study. J Bone Joint Surg Am 1978;60:1–6. 624747

[14] Richards RR An KN Bigliani LU A standardized method for the assessment of shoulder function. J Shoulder Elbow Surg 1994;3:347–52. 22958838 10.1016/S1058-2746(09)80019-0

[15] Throckmorton T Kuhn JE Fractures of the medial end of the clavicle. J Shoulder Elbow Surg 2007;16:49–54. 17169583 10.1016/j.jse.2006.05.010

[16] Bartonícek J Fric V Pacovský V Displaced fractures of the medial end of the clavicle: report of five cases. J Orthop Trauma 2010;24:e31–5. 20335748 10.1097/BOT.0b013e3181aa5505

[17] Partal G Meyers KN Sama N Superior versus anteroinferior plating of the clavicle revisited: a mechanical study. J Orthop Trauma 2010;24:420–5. 20577072 10.1097/BOT.0b013e3181c3f6d4

